# Celebrating with the ‘beetle’ man: Terry Erwin’s 75^th^ birthday

**DOI:** 10.3897/zookeys.541.7316

**Published:** 2015-12-01

**Authors:** Pensoft Editorial Office

**Affiliations:** 1 Pensoft Publishers, Sofia, Bulgaria

Our much respected Editor-in-Chief turns 75 years on 1^st^ December, and we would like to seize the opportunity to thank him for all the support and wish him many more years of success and fruitful collaboration with us. Terry Erwin was with ZooKeys from the very beginning. Words can barely describe how much has he contributed to strengthening and developing the journal, especially during its infancy. ZooKeys wouldn’t be what it is now without his great enthusiasm, endless care and friendly attitude to our staff (see our latest editorial Erwin T, Stoev P, Georgiev T, Penev L (2015) ZooKeys 500: traditions and innovations hand-in-hand servicing our taxonomic community). With the following short biographic note, we would like to wholeheartedly say to our god father

## Thank you, Terry! We wish you a long, healthy and happy life!

Terry L. Erwin was born in St. Helena of Napa County, California on December 1, 1940. His father was a “tin-knocker” i.e., a sheetmetal worker, and race car driver in the California circuit; his mother was a government clerk. Terry spent his youth trout fishing in the High Sierra with his maternal grandfather. As a teenager, with prodding from his father, he began building hot-rod cars and was a founding member and later the President of the California Conquistadores, a hot-rod club in the San Francisco Bay Area.

**Figure:**
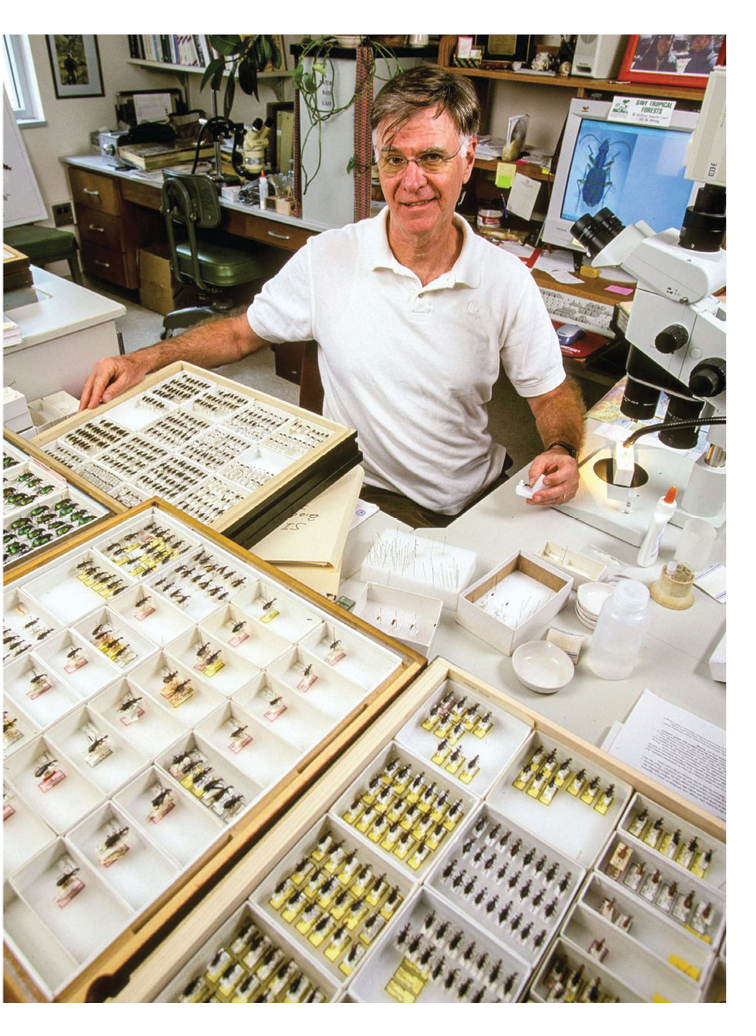
Terry Erwin

He put himself through college by working on Atomic Submarines at Mare Island Naval Shipyard where he was a helper in the “Asbestos” department. It was exactly at this time, when he made the life-changing decision that following his father’s footsteps was not in the cards for him. He then became a student under the guidance of the noted Coleopterist, J. Gordon Edwards, at San Jose State College. With the help of Gordon’s enthusiasm and guidance, Terry solved the intractable taxonomy of the Californian Bombardier Beetles – a group that still stays close to his heart. He then went on to explore the global bombardian beetle fauna for his dissertation under the mentorship of George E. Ball at the University of Alberta, Canada, finishing in 1969, who to this day is still his dear Mentor. And, continuing Ball’s tradition of mentoringship, he has now his own Mentees, all young women working on carabid beetle projects.

Having decided that he wanted to work under the three greatest (living) carabidologists, the first being Prof. Ball, Terry obtained postdoctoral fellowships at the Museum of Comparative Zoology at Harvard with Philip J. Darlington Jr. and at Lund University, Sweden with Carl H. Lindroth. However, a position opened at the National Museum of Natural History, Smithsonian Institution (then the USNM or United States National Museum) in the Department of Entomology upon the retirement of Oscar Cartwright. With the support of Washington Biologists Field Club (WBFC) members Paul Spangler (Coleopterist) and Karl Krombien (Chairman of Entomology), Terry was able to accept the position and within two months took a year-long sabbatical to Sweden to study with Lindroth.

He returned in 1971, to take up the reins as the second Coleopterist within the Department (now he is the only Coleopterist with the Smithsonian Institution in charge of 12 million museum specimens). While in Sweden, the Chairmanship of the Department turned over from Karl Krombien to Paul D. Hurd, who upon arrival saw on his new desk a proposal previously left by Terry asking to obtain funding for studies of the California carabid beetles. Having learned of monies for studies in Central America, Hurd crossed out “California” and wrote “Panama.” To Terry’s great surprise, he arrived home from Sweden only to find out he was checked in on the next plane to the Canal Zone. That journey made the beginning of a lifetime career devoted to the studies of biodiversity in Neotropical forests.

In 1982, with the publication of a small paper (Erwin TL (1982) Tropical forests: Their richness in Coleoptera and other arthropod species. The Coleopterists Bulletin 36 (1): 74–75) on the beetle fauna of a Panamanian tree species, Terry created a cottage industry in canopy studies both with the fogging technique of collecting canopy arthropods, but also in trying to estimate the number of species on the planet. In that paper, he had hypothesized that there were as many as 30 million species worldwide, an order of magnitude difference from the existing estimates at the time predicting just 1 million. A lot of people got excited, especially those in the Conservation business. If true, this meant that the world was losing a lot more species than previously imagined. The tiny 1.5-page paper that Terry wrote 34 years ago, has now been cited more than 1000 times.

Today, Terry continues his studies of biodiversity in the western Amazon Basin, at present in Ecuador. He has also not forgotten his favorite beetles, currently conducting taxonomic studies on carabids, particularly those genera living in the rainforest canopy. Being a prolific author he has written altogether (as of 20 November 2015) 270 papers, books, and monographs, most of them dealing with his favorite coleopterans. Keep up with Terry's publications on Google Scholars.

More information about Terry Erwin and his exciting career can be found in Rice ME (2015) Terry L. Erwin: She Had a Black Eye and in Her Arm She Held a Skunk. American Entomologist, volume 61, number 1, republished in ZooKeys 500: 9–24. doi: 10.3897/zookeys.500.9772.

**Table 1.** List of taxa named after Terry Erwin.

Order	Family	Subfamily (1)	Genus (2)	Species (47)	Subspecies (1)	Specauthor	Specdate	Locality
Coleoptera	Carabidae		*Erwiniana*			Paulsen & Smith	2003-	Neotropics
Hymenoptera	Dryinidae	Erwininae	*Erwinius*			Olmi & Guglielmino	2010-	Ecuador
Coleoptera	Carabidae		*Abaris*	*erwini*		Will	2002-	Bolivia; Perú
Coleoptera	Carabidae		*Anaulacus*	*erwini*		Ball & Shpeley	2002-	Perú
Coleoptera	Carabidae		*Anillinus*	*erwini*		Sokolov & Carlton	2004-	USA - NC, VA
Coleoptera	Carabidae		*Bembidion*	*erwini*		Perrault	1982-	México - OA
Coleoptera	Carabidae		*Calathus*	*erwini*		Ball & Nègre	1972-	México - JA, MH
Coleoptera	Carabidae		*Carabus*	*erwini*		Mandl	1975-	
Coleoptera	Carabidae		*Catascopus*	*erwini*		Straneo	1994-	
Coleoptera	Carabidae		*Clinidium*	*erwini*		Bell & Bell	2009-	Costa Rica
Coleoptera	Carabidae		*Coptodera*	*erwini*		Shpeley & Ball	1993-	Perú
Coleoptera	Carabidae		*Ctenostoma*	*erwini*		Naviaux	1998-	Panamá
Coleoptera	Carabidae		*Ctenostoma*	*erwini*		Naviaux	1998-	Ecuador - Galápagos Islands: Baltra (Seymour), Darwin (Culpepper), Fernandina (Narborough), Floreana, Genovesa (Tower), Pinta (Abingdon), Santa Cruz (Indefatigable), Santa Fé (Barrington), Wolf (Wenman).
	Carabidae		*Dyschiriodes*	*erwini*		Bulirsch	2009-	Neotropics
Coleoptera	Carabidae		*Dyschirius*	*erwini*		Bulirsch	2009-	Argentina – Santiago del Estero
Coleoptera	Carabidae		*Geocharidius*	*erwini*		Sokolov & Kavanugh	2014-	Central America
Coleoptera	Carabidae		*Eucheila*	*erwini*		Ball and Shpeley	2001-	Perú
Coleoptera	Carabidae		*Eurycoleus*	*erwini*		Spheley & Ball	2001-	Costa Rica
Coleoptera	Carabidae		*Paratrechus*	*erwini*		Barr	1982-	México – VC
Coleoptera	Carabidae		*Tachys*	*erwini*		Reichardt	1976-	Ecuador - Galápagos Islands: Baltra (Seymour), Darwin (Culpepper), Fernandina (Narborough), Floreana, Genovesa (Tower), Pinta (Abingdon), Santa Cruz (Indefatigable), Santa Fé (Barrington), Wolf (Wenman).
Coleoptera	Carabidae		*Tetracha*	*erwini*		Naviaux	2010-	Brazil - Roraima
Coleoptera	Carabidae		*Trichopsida*	*erwini*		Larochelle & Lariviere	2013-	New Zealand
Coleoptera	Carabidae		*Phloeoxena*	*megalops*	*erwinorum*	Ball	1975-	
Coleoptera	Carabidae		*Trichopselaphus*	*erwinorum*		Ball	1978-	Costa Rica; Panamá
Araneoidea	Araneidae		*Kaira*	*erwini*		Levi	1993-	Peru
Araneoidea	Oonopidae		*Gradunguloonops*	*erwini*		Grismado, Izquierdo, Gonzalez & Ramirez	2015-	Neotropics
Araneoidea	Oonopidae		*Dysderina*	*erwini*		Platnick, Berniker & Bonaldo	2013-	Neotropics
Araneoidea	Pisauridae		*Architis*	*erwini*		Santos	2007-	Neotropics
Coleoptera	Chrysomelidae		*Chrysomila*	*erwini*		Salvini, Escalona & /Furth	2008-	Peru
Coleoptera	Ciidae		*Phellinocis*	*erwini*		Lopes-Andrade & Lawrence	2005-	Neotropics
Coleoptera	Cleridae		*Enoclerus*	*erwini*		Ekis	1978-	Costa Rica, Panama
Coleoptera	Curculionidae		*Ceutorhynchus*	*erwini*		Korotyaev & O’Brien	2008-	USA - CA
Diptera	Aulacigastridae		*Aulacigraster*	*erwini*		Rung & Mathis	2011-	Ecuador
Diptera	Clusiidae		*Sobarocephala*	*erwini*		Londdale & Marshall	2012-	Neotropics
Diptera	Lauxaniidae		*Eurystratiomyia*	*erwini*		Gaimari & Silva	2010-	
Diptera	Tephritidae		*Molynocoelia*	*erwini*		Norrbom	2011-	Ecuador
Hemiptera	Cicadellidae		*Gabrita*	*erwini*		Nielson	2010-	Neotropics
Hemiptera	Cicadellidae		*Daedaloscarta*	*erwini*		Cavichioli & Takiya	2012-	Neotropics
Hemiptera	Cixiidae		*Loisirella*	*erwini*		Holzinger, Holzinger & Egger	2013-	Ecuador
Hemiptera	Rhyparochromidae		*Villalobosothignus*	*erwini*		Dellape & Montemayor	2011-	Ecuador
Hymenoptera	Aphelininae		*Punkaphytis*	*erwini*		Kim & Heraty	2012-	Neotropics
Hymenoptera	Dryinidae		*Deinodryinus*	*erwini*		Olmi	2008-	Neotropics
Hymenoptera	Eucharitidae		*Orasema*	*erwini*		Burks, Mottern & Heraty	2015-	Neotropics
Hymenoptera	Eurytomidae		*Khamul*	*erwini*		Gates	2008-	Ecuador
Hymenoptera	Ichneumonidae		*Stethantyx*	*erwini*		Townes	1971-	Neotropics
Hymenoptera	Trichogrammatidae		*Adryas*	*erwini*		Pinto & Owen	2004-	Neotropics
Psocoptera	Lachesillidae		*Waoraniella*	*erwini*		Garcia Aldrete	2006-	Ecuador
Psocoptera	Ptiloneuridae		*Loneura*	*erwini*		New & Thornton	1988-	Peru
Psocoptera	Ptiloneuridae		*Triplocania*	*erwini*		Neto, Rafael & Garcia Aldrete	2014-	Ecuador
Psocoptera	Psocidae		*Trichadenotecnum*	*erwini*		Yoshizawa, Garcia Aldrete & Mockford	2008-	Neotropics
Psocoptera	Troctopsocoidae		*Troctopsocoides*	*erwini*		Mockford & Garcia Aldrete	2014-	Peru

**Table 2.** List of the tribes described by Terry Erwin.

Tribe	Tribauthor	Tribdate
Systolosomatini	Erwin	1985:467
Amarotypini	Erwin	1985:467
Loxandrini	Erwin & Sims	1984:383
Xenaroswellanini	Erwin	2007:563

**Table 3.** List of the genera described by Terry Erwin.

Genus	Genauthor	Gendate
*Archaeocindis*	Kavanaugh & Erwin	1991:359
*Argentinatachoides*	Sallenave, Erwin & Roig	2008:8
*Bembidarenas*	Erwin	1972:8
*Costitachys*	Erwin	1974:128
*Geballusa*	Erwin	1994:574
*Gouleta*	Erwin	1994:578
*Guatemalteca*	Erwin	2004:12
*Guyanemorpha*	Erwin	2013:14
*Hybopteroides*	Erwin & Ball	2012:191
*Inpa*	Erwin	1978:31
*Manumorpha*	Erwin & Geraci	2008:86
*Meotachys*	Erwin	1974:130
*Moirainpa*	Erwin	1984:512
*Peruphorticus*	Erwin & Zamorano	2014:26
*Pseudophorticus*	Erwin	2004:7
*Quammenis*	Erwin	2000:279
*Samiriamorpha*	Erwin & Geraci	2008:89
*Tachysbembix*	Erwin	2004:3
*Tuxtlamorpha*	Erwin & Geraci	2008:82
*Valeriaaschero*	Erwin	2004:14
*Xenaroswelliana*	Erwin	2007:563
*Yasunimorpha*	Erwin & Geraci	2008:87

**Table 4.** List of the species described by Terry Erwin.

Genus	Species	Specauthor	Specdate	Locality
*Agra*	*aeroides*	Erwin	1983:284	Brazil
*Agra*	*anthrax*	Erwin	1986:314	Brazil
*Agra*	*ariasi*	Erwin	1982:200	Brazil
*Agra*	*atlas*	Erwin	1984:25	Trinidad
*Agra*	*atriperna*	Erwin	1984:42	Costa Rica; Panamá
*Agra*	*azureipennis*	Erwin	1982:208	Perú; Venezuela
*Agra*	*belize*	Erwin	1984:41	Belize
*Agra*	*blumax*	Erwin	1983:283	Brazil
*Agra*	*cachimbo*	Erwin	1984:30	Brazil
*Agra*	*cadabra*	Erwin	1986:303	Ecuador
*Agra*	*calamitas*	Erwin	1986:314	Brazil
*Agra*	*caliga*	Erwin	1982:206	Panamá
*Agra*	*campana*	Erwin	1983:273	Costa Rica; Panamá
*Agra*	*cauca*	Erwin	1998:509	Colombia
*Agra*	*cavei*	Erwin	1984:33	Paraguay
*Agra*	*chapada*	Erwin	1987:148	Brazil
*Agra*	*chocha*	Erwin	1986:304	Guyane; Suriname; Trinidad; Venezuela
*Agra*	*cobra*	Erwin	1982:56	Guyana
*Agra*	*coleps*	Erwin	1982:52	Guyane
*Agra*	*constans*	Erwin	1984:28	Brazil
*Agra*	*cuneolus*	Erwin	1983:272	Brazil
*Agra*	*cyaneucnemes*	Erwin	1984:45	Colombia
*Agra*	*dation*	Erwin	1987:156	Brazil
*Agra*	*dora*	Erwin	1984:34	Perú
*Agra*	*dorazul*	Erwin	1984:44	Colombia
*Agra*	*dryas*	Erwin	1982:55	Brazil
*Agra*	*ecaligis*	Erwin	1982:197	Ecuador
*Agra*	*ega*	Erwin	1982:60	Brazil
*Agra*	*eowilsoni*	Erwin	1998:509	Colombia
*Agra*	*eucera*	Erwin	1984:21	Panamá
*Agra*	*eucnemes*	Erwin	1984:23	Ecuador
*Agra*	*falsisagax*	Erwin	1982:55	Brazil
*Agra*	*fortuna*	Erwin	1983:270	Costa Rica; Panamá
*Agra*	*goyazella*	Erwin	1984:30	Brazil
*Agra*	*howdenorum*	Erwin	1982:194	Trinidad
*Agra*	*imaginis*	Erwin	1986:306	Brazil
*Agra*	*inca*	Erwin	1986:307	Perú
*Agra*	*inpa*	Erwin	1983:282	Brazil
*Agra*	*invicta*	Erwin	1982:58	Brazil
*Agra*	*iota*	Erwin	1984:33	Perú
*Agra*	*iquitosana*	Erwin	1982:201	Brazil; Perú
*Agra*	*lata*	Erwin	1987:157	Bolivia
*Agra*	*limulus*	Erwin	1982:199	Ecuador; Perú
*Agra*	*littleorum*	Erwin	1984:28	Brazil
*Agra*	*luehea*	Erwin	1983:285	Panamá
*Agra*	*magdalena*	Erwin	1987:159	Colombia
*Agra*	*maxli*	Erwin	1982:69	Brazil
*Agra*	*memnon*	Erwin	1987:157	Brazil
*Agra*	*mniszechi*	Erwin	1982:64	Guyane
*Agra*	*moira*	Erwin	1983:288	Brazil
*Agra*	*nigrarima*	Erwin	1984:25	Trinidad
*Agra*	*nola*	Erwin	1986:302	Costa Rica; Ecuador; Panamá
*Agra*	*notichlora*	Erwin	1984:33	Paraguay
*Agra*	*notiocyanea*	Erwin	1984:34	Bolivia
*Agra*	*nox*	Erwin	1984:25	Guyane
*Agra*	*oiapoquensis*	Erwin	1982:53	Brazil
*Agra*	*orabrocha*	Erwin	1984:43	Panamá
*Agra*	*paloma*	Erwin	1984:39	México – OA
*Agra*	*para*	Erwin	1987:152	Brazil
*Agra*	*pearsoni*	Erwin	1984:36	Perú
*Agra*	*pennyi*	Erwin	1982:64	Brazil
*Agra*	*perkinsorum*	Erwin	1986:298	México – GO, JA, MX, NA
*Agra*	*phainops*	Erwin	1986:306	Surinam; Venezuela
*Agra*	*phite*	Erwin	1987:149	Bolivia
*Agra*	*pseuderythropus*	Erwin	1982:52	Guyane
*Agra*	*quararibea*	Erwin	1993:25	Perú
*Agra*	*rhomboides*	Erwin	1982:60	Guyane
*Agra*	*rubra*	Erwin	1987:153	Brazil
*Agra*	*rufarima*	Erwin	1984:27	Trinidad
*Agra*	*saltatrix*	Erwin	1982:65	Perú
*Agra*	*saramax*	Erwin	1993:22	Bolivia; Ecuador
*Agra*	*sasquatch*	Erwin	1982:208	Brazil
*Agra*	*satipo*	Erwin	1984:34	Perú
*Agra*	*seabrae*	Erwin	1982:205	Brazil
*Agra*	*serra*	Erwin	1984:28	Brazil
*Agra*	*sironyx*	Erwin	1984:25	Trinidad
*Agra*	*sphenarion*	Erwin	1982:65	Perú
*Agra*	*spina*	Erwin	1983:272	Brazil
*Agra*	*stockwelli*	Erwin	1984:36	Panamá
*Agra*	*tarapoto*	Erwin	1984:30	Perú
*Agra*	*tarapotoana*	Erwin	1982:201	Perú
*Agra*	*tessera*	Erwin	1983:272	Brazil
*Agra*	*tingomaria*	Erwin	1984:36	Perú
*Agra*	*titan*	Erwin	1982:201	Brazil; Guyane
*Agra*	*tuitis*	Erwin	1987:156	Perú
*Agra*	*tumatumari*	Erwin	1982:199	Guyana
*Agra*	*varzeicola*	Erwin	1982:199	Brazil
*Agra*	*vate*	Erwin	1986:302	Costa Rica; Guatemala; México – VC; Panamá
*Agra*	*vation*	Erwin	1983:287	Perú
*Agra*	*vesedes*	Erwin	1984:46	South America
*Agra*	*xingu*	Erwin	1984:28	Brazil
*Agra*	*yeti*	Erwin	1982:207	Brazil
*Agra*	*yoda*	Erwin	1982:64	Guyane
*Agra*	*zona*	Erwin	1983:282	Panamá
*Agra*	*demerasae*	Erwin	1984:23	Guyana
*Agra*	*itatiaya*	Erwin	1986:312	Brazil
*Agra*	*kayae*	Erwin	1984:39	Costa Rica; Belize; Guatemala; Honduras; México – CS, OA, TA; Panamá
*Agra*	*lavernae*	Erwin	1978:265	Costa Rica; Panamá
*Agra*	*olivencana*	Erwin	1982:201	Brazil
*Agra*	*perinvicta*	Erwin	1982:56	Brazil
*Agra*	*yodella*	Erwin	1982:197	Guyane
*Agra*	*tingo*	Erwin	2000:103	Perú
*Agra*	*biolat*	Erwin	2000:104	Perú
*Agra*	*aeris*	Erwin	2000:104	Perú
*Agra*	*solimoes*	Erwin	2000:105	Brazil
*Agra*	*conhormigas*	Erwin	2000:106	Perú
*Agra*	*lilu*	Erwin	2000:108	Brazil
*Agra*	*servatorum*	Erwin	2000:110	Perú
*Agra*	*lindae*	Erwin	2000:111	Perú
*Agra*	*rondonia*	Erwin	2000:112	Brazil
*Agra*	*nex*	Erwin	2000:112	Brazil
*Agra*	*manu*	Erwin	2000:112	Perú
*Agra*	*dax*	Erwin	2000:115	Panamá
*Agra*	*orinocensis*	Erwin	2000:8	Venezuela
*Agra*	*novaurora*	Erwin	2000:9	Ecuador
*Agra*	*alinahui*	Erwin	2000:9	Ecuador
*Agra*	*superba*	Erwin	2000:10	Brazil; Venezuela
*Agra*	*maracay*	Erwin	2000:13	Venezuela
*Agra*	*bci*	Erwin	2000:14	Costa Rica; Panamá
*Agra*	*falcon*	Erwin	2000:14	Venezuela
*Agra*	*hovorei*	Erwin	2000:15	México – VC
*Agra*	*tuxtlas*	Erwin	2000:15	México – VC
*Agra*	*zapotal*	Erwin	2000:15	Guatemala; México – CS, VC
*Agra*	*hespenheide*	Erwin	2000:16	Costa Rica
*Agra*	*paratax*	Erwin	2000:16	Costa Rica
*Agra*	*samiria*	Erwin	2000:16	Perú
*Agra*	*duckworthorum*	Erwin	2000:16	Panamá
*Agra*	*eponine*	Erwin	2000:16	Costa Rica
*Agra*	*inbio*	Erwin	2000:17	Costa Rica
*Agra*	*pichincha*	Erwin	2000:17	Ecuador
*Agra*	*othello*	Erwin	2000:19	Ecuador
*Agra*	*smurf*	Erwin	2000:19	Brazil
*Agra*	*magnifica*	Erwin	2000:19	Perú
*Agra*	*suprema*	Erwin	2000:20	Brazil
*Agra*	*yola*	Erwin	2000:282	Costa Rica
*Agra*	*mime*	Erwin	2000:258	Ecuador
*Agra*	*dable*	Erwin	2002:37	Costa Rica
*Agra*	*liv*	Erwin	2002:39	Costa Rica; Panamá
*Agra*	*santarosa*	Erwin	2002:40	Costa Rica
*Agra*	*solisi*	Erwin	2002:41	Costa Rica
*Agra*	*jimwappes*	Erwin	2002:16	Costa Rica
*Agra*	*winnie*	Erwin	2002:17	Costa Rica; El Salvador
*Agra*	*giesberti*	Erwin	2002:19	Costa Rica; Guatemala; Panamá
*Agra*	*not*	Erwin	2002:21	Costa Rica
*Agra*	*phallica*	Erwin	2002:22	Costa Rica
*Agra*	*turrialba*	Erwin	2002:24	Costa Rica
*Agra*	*catie*	Erwin	2002:27	Costa Rica
*Agra*	*fugax*	Erwin	2002:28	Costa Rica
*Agra*	*sirena*	Erwin	2002:29	Costa Rica
*Agra*	*quesada*	Erwin	2002:31	Costa Rica
*Agra*	*janzeni*	Erwin	2002:33	Costa Rica
*Agra*	*katewinsletae*	Erwin	2002:35	Costa Rica
*Agra*	*ubicki*	Erwin	2002:43	Costa Rica
*Agra*	*granodeoro*	Erwin	2002:45	Costa Rica
*Agra*	*schwarzeneggeri*	Erwin	2002:46	Costa Rica
*Agra*	*solanoi*	Erwin	2002:48	Costa Rica
*Agra*	*notcatie*	Erwin	2002:50	Costa Rica
*Agra*	*pitilla*	Erwin	2002:53	Costa Rica
*Agra*	*catbellae*	Erwin	2002:56	Costa Rica
*Agra*	*delgadoi*	Erwin	2002:58	Costa Rica; Panamá
*Agra*	*julie*	Erwin	2002:59	Costa Rica
*Agra*	*ichabod*	Erwin	2002:61	Costa Rica
*Agra*	*monteverde*	Erwin	2002:62	Costa Rica
*Agra*	*zumbado*	Erwin	2002:63	Costa Rica
*Agra*	*zuniga*	Erwin	2002:65	Costa Rica
*Agra*	*cruciaria*	Erwin	2010:6	Brazil
*Agra*	*grace*	Erwin	2010:7	Peru
*Agra*	*max*	Erwin	2010:9	Brazil
*Agra*	*minasianus*	Erwin	2010:10	Brazil
*Agra*	*notpusilla*	Erwin	2010:11	Brazil
*Agra*	*pseudopusilla*	Erwin	2010:13	Brazil
*Agra*	*ce*	Erwin	2010:16	Peru
*Agra*	*maia*	Erwin	2010:17	Bolivia
*Agra*	*piranha*	Erwin	2010:18	Ecuador
*Agra*	*risseri*	Erwin	2010:19	Bolivia
*Agra*	*tiputini*	Erwin	2010:23	Ecuador
*Apotomus*	*reichardti*	Erwin	1980:100	Brazil – MATO GROSSO
*Argentinatachoides*	*balli*	Sallenave, Erwin & Roig	2008:8	Argentina – La Rioja, Mendoza
*Asklepia*	*campbellorum*	Zamorano & Erwin	2014:39	Brazil
*Asklepia*	*demiti*	Erwin & Zamorano	2014:41	Brazil
*Asklepia*	*duofos*	Zamorano & Erwin	2014:43	Brazil
*Asklepia*	*grammechrysea*	Zamorano & Erwin	2014:44	Perú
*Asklepia*	*laetitia*	Zamorano & Erwin	2014:49	Colombia
*Asklepia*	*matomena*	Zamorano & Erwin	2014:52	Brazil
*Asklepia*	*adisi*	Erwin & Zamorano	2014:55	Brazil
*Asklepia*	*asuncionensis*	Erwin & Zamorano	2014:57	Paraguay
*Asklepia*	*biolat*	Erwin & Zamorano	2014:58	Perú
*Asklepia*	*bracheia*	Zamorano & Erwin	2014:60	Perú
*Asklepia*	*cuiabaensis*	Erwin & Zamorano	2014:62	Brazil
*Asklepia*	*ecuadoriana*	Erwin & Zamorano	2014:64	Ecuador
*Asklepia*	*kathleenae*	Erwin & Zamorano	2014:66	Brazil
*Asklepia*	*macrops*	Erwin & Zamorano	2014:67	Argentina
*Asklepia*	*marchantaria*	Erwin & Zamorano	2014:69	Brazil
*Asklepia*	*marituba*	Zamorano & Erwin	2014:71	Brazil
*Asklepia*	*pakitza*	Erwin & Zamorano	2014:72	Perú
*Asklepia*	*paraguayensis*	Zamorano & Erwin	2014:74	Paraguay
*Asklepia*	*samiriaensis*	Zamorano & Erwin	2014:77	Perú
*Asklepia*	*stalametlitos*	Zamorano & Erwin	2014:79	Bolivia
*Asklepia*	*surinamensis*	Zamorano & Erwin	2014:81	Surinam
*Asklepia*	*vigilante*	Erwin & Zamorano	2014:85	Perú
*Aspasiola*	*bonita*	Erwin	2004:9	Costa Rica
*Aspasiola*	*osa*	Erwin	2004:14	Costa Rica
*Aspasiola*	*selva*	Erwin	2004:16	Costa Rica
*Aspasiola*	*steineri*	Erwin	2004:18	Costa Rica
*Badister*	*amazonus*	Erwin & Ball	2011:409	Perú
*Bembidion*	*palosverdes*	Kavanaugh & Erwin	1992:312	USA – CA
*Bembidion*	*aeger*	Erwin	1982:475	Costa Rica
*Bembidion*	*armuelles*	Erwin	1982:481	Panamá
*Bembidion*	*barrense*	Erwin	1982:482	Panamá
*Bembidion*	*chiriqui*	Erwin	1982:476	Panamá
*Bembidion*	*cortes*	Erwin	1982:480	Honduras; México
*Bembidion*	*diabola*	Erwin	1982:473	Costa Rica
*Bembidion*	*edwardsi*	Erwin	1982:475	Costa Rica
*Bembidion*	*franiae*	Erwin	1982:479	Guatemala
*Bembidion*	*ixtatan*	Erwin	1982:484	Guatemala
*Bembidion*	*lavernae*	Erwin	1982:472	Costa Rica
*Bembidion*	*nahuala*	Erwin	1982:477	Guatemala
*Bembidion*	*purulha*	Erwin	1982:466	Guatemala
*Bembidion*	*quetzal*	Erwin	1982:473	Guatemala
*Brachinus*	*aabaaba*	Erwin	1970:161	México – SL; USA – KS, NM, TX
*Brachinus*	*adustipennis*	Erwin	1969:287	Costa Rica; CUBA; MÉXICO – AG, NA, SI, SO, TA, TM, VC, YC; PANAMÁ; USA – AL, AR, FL, GA, IL, IN, KS, LA, MA, MI, MO, MS, NM, NY, OK, SC, TN, TX, VA, WI
*Brachinus*	*alexiguus*	Erwin	1970:57	USA – MS, OK, TX
*Brachinus*	*capnicus*	Erwin	1970:60	USA – NC
*Brachinus*	*chalchihuitlicue*	Erwin	1970:79	Costa Rica; GUATEMALA; MÉXICO – CM, GO, NA, SI; PANAMÁ
*Brachinus*	*chirriador*	Erwin	1970:80	Honduras; México – CS, JA, NA, SL, TM, VC
*Brachinus*	*cibolensis*	Erwin	1970:98	México – DU; USA – AZ, NM
*Brachinus*	*cyanochroaticus*	Erwin	1969:283	Canada – BC, MB, NB, ON, PQ, SK; USA – CO, CT, IA, ID, IL, IN, KS, MA, ME, MI, MN, MO, MT, ND, NE, NH, NJ, NY, OH, PA, SD, VT, WI, WY
*Brachinus*	*explosus*	Erwin	1970:161	México – SL; USA – AZ
*Brachinus*	*favicollis*	Erwin	1965:11	México – BJ; USA – AZ, CA
*Brachinus*	*fulminatus*	Erwin	1969:288	USA – CT, DE, IN, MA, MD, NC, NH, NJ, NY, PA, RI, VA, WI
*Brachinus*	*galactoderus*	Erwin	1970:132	México – GO, NA, OA, SI, SO
*Brachinus*	*gebhardis*	Erwin	1965:6	México – BJ; USA – AZ, CA
*Brachinus*	*ichabodopsis*	Erwin	1970:150	USA – FL
*Brachinus*	*imperialensis*	Erwin	1965:17	México – DU, SI, SL, SO; USA – AZ, CA, CO, NM, NV, TX
*Brachinus*	*imporcitis*	Erwin	1970:114	USA – AZ
*Brachinus*	*javalinopsis*	Erwin	1970:109	USA – AZ, NM, TX
*Brachinus*	*kavanaughi*	Erwin	1969:287	México – NL, TM; USA – CO, IL, KS, MO, MT, NE, NY, OH, OK, SD, TX, WI, WY
*Brachinus*	*microamericanus*	Erwin	1969:287	USA – KY, LA, MI, MO, MS
*Brachinus*	*mobilis*	Erwin	1970:159	USA – AL
*Brachinus*	*oaxacensis*	Erwin	1970:117	México – GO, OA
*Brachinus*	*pallidus*	Erwin	1965:8	USA – CA, OR, WA
*Brachinus*	*sonorous*	Erwin	1970:163	México – SI, SO
*Brachinus*	*velutinus*	Erwin	1965:17	USA – CA
*Brachinus*	*vulcanoides*	Erwin	1969:287	USA – FL, MA, MD, NH, NJ, NY, RI
*Chelonodema*	*inbio*	Erwin	2000:281	Costa Rica
*Coptocarpus*	*philipi*	Erwin	1974:5	Australia
*Coptocarpus*	*chimbu*	Erwin	1974:7	New Guinea
*Coptocarpus*	*yorkensis*	Erwin	1974:7	Australia
*Coptocarpus*	*grossus*	Erwin	1974:8	Australia
*Costitachys*	*inusitatus*	Erwin	1974:130	Brazil; Guyane; Trinidad
*Costitachys*	*tena*	Erwin & Kavanaugh	2006:335	Ecuador
*Epikastea*	*biolat*	Erwin	2004:7	Perú
*Epikastea*	*grace*	Erwin	2004:13	Perú
*Epikastea*	*mancocapac*	Erwin	2004:15	Perú
*Epikastea*	*piranha*	Erwin	2004:16	Ecuador
*Epikastea*	*poguei*	Erwin	2004:17	Perú
*Erwiniana*	*aetholia*	(Erwin)	1973:12	Bolivia
*Erwiniana*	*alticola*	(Erwin)	1994:614	Colombia
*Erwiniana*	*am*	(Erwin)	1994:594	Perú
*Erwiniana*	*anchicaya*	(Erwin)	1994:607	Colombia
*Erwiniana*	*angustia*	(Erwin)	1994:592	Perú
*Erwiniana*	*anterocostis*	(Erwin)	1973:14	Costa Rica
*Erwiniana*	*apicisulcata*	(Erwin)	1973:18	Costa Rica
*Erwiniana*	*baeza*	(Erwin)	1994:582	Ecuador
*Erwiniana*	*batesi*	(Erwin)	1973:19	Brazil
*Erwiniana*	*bisulcifrons*	(Erwin)	1973:27	Brazil
*Erwiniana*	*chiriboga*	(Erwin)	1994:616	Ecuador
*Erwiniana*	*crassa*	(Erwin)	1994:583	Perú
*Erwiniana*	*dannyi*	(Erwin)	1994:616	Ecuador
*Erwiniana*	*depressisculptilis*	(Erwin)	1994:602	Ecuador
*Erwiniana*	*equanegrei*	(Erwin)	1994:586	Ecuador
*Erwiniana*	*esheje*	(Erwin)	1994:584	Perú
*Erwiniana*	*eugeneae*	(Erwin)	1994:605	Colombia; Perú
*Erwiniana*	*exigupunctata*	(Erwin)	1994:608	Brazil; Ecuador; Perú
*Erwiniana*	*foveosculptilis*	(Erwin)	1994:604	Brazil
*Erwiniana*	*grossipunctata*	(Erwin)	1973:20	Brazil
*Erwiniana*	*hamatilis*	(Erwin)	1994:598	Ecuador
*Erwiniana*	*henryi*	(Erwin)	1994:582	Ecuador
*Erwiniana*	*huacamayas*	(Erwin)	1994:617	Ecuador
*Erwiniana*	*indetecticostis*	(Erwin)	1994:595	Ecuador
*Erwiniana*	*iris*	(Erwin)	1973:11	Bolivia; Perú
*Erwiniana*	*irisculptilis*	(Erwin)	1994:603	Ecuador
*Erwiniana*	*jacupiranga*	(Erwin)	1994:613	Brazil
*Erwiniana*	*jefe*	(Erwin)	1994:608	Panamá
*Erwiniana*	*manusculptilis*	(Erwin)	1994:586	Perú
*Erwiniana*	*misahualli*	(Erwin)	1994:602	Ecuador
*Erwiniana*	*negrei*	(Erwin)	1973:12	Venezuela
*Erwiniana*	*nigripalpis*	(Erwin)	1973:9	Panamá
*Erwiniana*	*notesheje*	(Erwin)	1994:584	Ecuador; Perú
*Erwiniana*	*notparkeri*	(Erwin)	1994:598	Colombia
*Erwiniana*	*nox*	(Erwin)	1994:596	Ecuador
*Erwiniana*	*para*	(Erwin)	1994:589	Brazil
*Erwiniana*	*parainsularis*	(Erwin)	1973:26	Venezuela
*Erwiniana*	*parapara*	(Erwin)	1994:585	Brazil
*Erwiniana*	*parkeri*	(Erwin)	1994:597	Perú
*Erwiniana*	*pfunorum*	(Erwin)	1994:600	Brazil –; Ecuador; Perú
*Erwiniana*	*protosculptilis*	(Erwin)	1994:601	Perú
*Erwiniana*	*punctisculptilis*	(Erwin)	1994:605	Perú
*Erwiniana*	*quadrata*	(Erwin)	1994:600	Perú
*Erwiniana*	*rosebudae*	(Erwin)	1994:612	Bolivia; Ecuador
*Erwiniana*	*samiria*	(Erwin)	1994:593	Perú
*Erwiniana*	*seriata*	(Erwin)	1973:19	Brazil
*Erwiniana*	*wygo*	(Erwin)	1994:615	Colombia
*Eucamaragnathus*	*amapa*	Erwin & Stork	1985:440	Brazil – AMAPÁ
*Eucamaragnathus*	*jaws*	Erwin & Stork	1985:441	Brazil – PARANÁ
*Geballusa*	*microtreta*	Erwin	1973:24	Costa Rica; Panamá; Perú
*Geballusa*	*nannotreta*	Erwin	1994:577	Brazil
*Geballusa*	*oligotreta*	Erwin	1994:575	Panamá
*Geballusa*	*polytreta*	Erwin	1973:25	Brazil
*Geballusa*	*rex*	Erwin	1994:576	Brazil
*Geocharidius*	*gimlii*	Erwin	1982:488	Guatemala
*Geocharidius*	*phineus*	Erwin	1982:491	Guatemala
*Geocharidius*	*romeoi*	Erwin	1982:488	Guatemala
*Gouleta*	*gentryi*	Erwin	1994:580	Perú
*Gouleta*	*notiophiloides*	Erwin	1973:22	Brazil; Perú
*Gouleta*	*spangleri*	Erwin	1973:23	Panamá
*Guatemalteca*	*virgen*	Erwin	2004:12	Costa Rica; Guatemala; Guyane; Perú
*Halocoryza*	*whiteheadiana*	Erwin	2011:7	México – BJ
*Hyboptera*	*apollonia*	Erwin	2004:33	Costa Rica; Panamá
*Hyboptera*	*auxiliadora*	Erwin	2004:35	Costa Rica; México –; Panamá; USA –TX
*Hybopteroides*	*biolat*	Erwin & Ball	2012:195	Perú
*Hybopteroides*	*karolynae*	Erwin & Ball	2012:196	Perú
*Hybopteroides*	*penrosei*	Erwin & Ball	2012:198	Ecuador
*Inpa*	*psydroides*	Erwin	1978:31	Brazil –; Perú; Surinam
*Leistus*	*madmeridianus*	Erwin	1970:117	USA – CA
*Lionepha*	*chintimini*	(Erwin & Kavanaugh)	1981:63	USA – OR
*Lionepha*	*lindrothellus*	(Erwin & Kavanaugh)	1981:61	Canada – BC (VCI); USA – AK, WA
*Lionepha*	*lummi*	(Erwin & Kavanaugh)	1981:62	Canada – BC; USA – WA
*Loricera*	*aptena*	Ball & Erwin	1969:889	México – CH, DU, MH, SI
*Manumorpha*	*biolat*	Erwin & Geraci	2008:86	Perú
*Mizotrechus*	*poirieri*	Erwin	2011:110	Guyane
*Mizotrechus*	*bellorum*	Erwin	2011:88	Guyane
*Mizotrechus*	*woldai*	Erwin	2011:112	Panamá
*Mizotrechus*	*belvedere*	Erwin	2011:89	Guyane
*Mizotrechus*	*brulei*	Erwin	2011:90	Guyane
*Mizotrechus*	*chontalesensis*	Erwin	2011:92	Nicaragua
*Mizotrechus*	*costaricensis*	Erwin	2011:94	Costa Rica
*Mizotrechus*	*dalensi*	Erwin	2011:95	Guyane
*Mizotrechus*	*edithpiafae*	Erwin	2011:97	South America
*Mizotrechus*	*fortunensis*	Erwin	2011:98	Panamá
*Mizotrechus*	*gorgona*	Erwin	2011:100	Colombia
*Mizotrechus*	*grossus*	Erwin	2011:101	Guyane
*Mizotrechus*	*jefe*	Erwin	2011:101	Panamá
*Mizotrechus*	*marielaforetae*	Erwin	2011:104	Guyane
*Mizotrechus*	*minutus*	Erwin	2011:106	Guyane
*Mizotrechus*	*neblinensis*	Erwin	2011:108	Guyane; Venezuela
*Moirainpa*	*amazona*	Erwin	1984:512	Brazil –; Perú
*Moriosomus*	*motschulskyi*	Erwin & Moore	2007:4	Perú
*Nebria*	*piute*	Erwin & Ball	1972:95	USA – UT
*Nebria*	*piute*	Erwin & Ball	1972:95	USA – UT
*Nebria*	*piute*	Erwin & Ball	1972:95	USA – UT
*Paratachys*	*potomaca*	Erwin	1981:152	USA – MA, MD, NC, OH, PA, VA
*Pericompsus*	*alcimus*	Erwin	1974:72	Argentina – Formosa, Salta, Santiago del Estero; Bolivia; Paraguay; Perú
*Pericompsus*	*amygdali*	Erwin	1974:66	Bolivia; Venezuela
*Pericompsus*	*acon*	Erwin	1974:77	Bolivia
*Pericompsus*	*anassa*	Erwin	1974:77	Argentina – Tucumán; Paraguay
*Pericompsus*	*bilbo*	Erwin	1974:31	Venezuela
*Pericompsus*	*callicalymma*	Erwin	1974:68	Argentina – Buenos Aires; Brazil – Mato Grosso
*Pericompsus*	*carinatus*	Erwin	1974:93	Brazil – Mato Grosso
*Pericompsus*	*commotes*	Erwin	1974:31	Venezuela
*Pericompsus*	*crossarchon*	Erwin	1974:34	Brazil – Mato Grosso
*Pericompsus*	*crossodmos*	Erwin	1974:35	Argentina – Buenos Aires, Entre Ríos, Tucumán; Brazil – Rio Grande do Sul
*Pericompsus*	*crossotus*	Erwin	1974:85	Brazil – Mato Grosso; Paraguay
*Pericompsus*	*diabalius*	Erwin	1974:83	Colombia
*Pericompsus*	*dynastes*	Erwin	1974:28	Venezuela
*Pericompsus*	*eubothrus*	Erwin	1974:71	Brazil – Mato Grosso
*Pericompsus*	*gongylus*	Erwin	1974:37	México – CP
*Pericompsus*	*jamcubanus*	Erwin	1974:57	Cuba; Jamaica
*Pericompsus*	*leechi*	Erwin	1974:46	México – NA
*Pericompsus*	*leucocarenus*	Erwin	1974:34	El Salvador; México – MX, MH, SL, TM, VC
*Pericompsus*	*micropegasus*	Erwin	1974:75	Bolivia
*Pericompsus*	*morantensis*	Erwin	1974:61	Dominican Republic; Jamaica
*Pericompsus*	*nonandinus*	Erwin	1974:66	Brazil – Mato Grosso
*Pericompsus*	*pauli*	Erwin	1974:47	El Salvador; México – CS
*Pericompsus*	*pegasus*	Erwin	1974:75	Bolivia
*Pericompsus*	*philipi*	Erwin	1974:62	Cuba; Dominican Republic; Haiti
*Pericompsus*	*polychaetus*	Erwin	1974:88	Bolivia
*Pericompsus*	*prionomus*	Erwin	1974:63	Panamá
*Pericompsus*	*reticulatus*	Erwin	1974:24	Brazil – Ceará; Guyana
*Pericompsus*	*rorschachinus*	Erwin	1974:92	Bolivia; Perú; Venezuela
*Pericompsus*	*sagma*	Erwin	1974:45	México – CS, CM, GO, VC
*Pericompsus*	*silicis*	Erwin	1974:51	Brazil – Mato Grosso; Colombia; Costa Rica; Honduras
*Pericompsus*	*stenocitharus*	Erwin	1974:84	Paraguay
*Pericompsus*	*subincisus*	Erwin	1974:70	Brazil – Goiás, Mato Grosso
*Pericompsus*	*tetraphalarus*	Erwin	1974:68	Bolivia
*Pericompsus*	*tlaloc*	Erwin	1974:54	Costa Rica; México – CS, CM, JA, NA, OA, SL, SI, SO, VC
*Pericompsus*	*tolype*	Erwin	1974:39	Argentina – La Rioja, Salta; Bolivia; Brazil – Santa Catarina; Venezuela
*Peruphorticus*	*gulliveri*	Erwin & Zamorano	2014:26	Ecuador; Perú
*Polyderis*	*antiqua*	Erwin	1971:234	México –
*Polyderis*	*moira*	Erwin	1984:513	Brazil
*Polyderis*	*nympha*	Erwin	1984:515	Brazil –; Perú
*Polyderis*	*terra*	Erwin	1984:514	Brazil
*Polyderis*	*ucayali*	Erwin	1984:514	Brazil –; Perú
*Pseudomorpha*	*santarita*	Erwin & Amundson	2013:47	USA – AZ, NM
*Pseudomorpha*	*santacruz*	Erwin & Amundson	2013:45	USA – AZ
*Pseudomorpha*	*patagonia*	Erwin & Amundson	2013:39	USA – AZ
*Pseudomorpha*	*huachinera*	Amundson & Erwin	2013:36	México – SO; USA – AZ
*Pseudomorpha*	*penablanca*	Amundson & Erwin	2013:42	USA – AZ
*Pseudomorpha*	*pima*	Amundson & Erwin	2013:44	USA – AZ
*Pseudophorticus*	*puncticollis*	Erwin	2004:8	Costa Rica
*Quammenis*	*spectabilis*	Erwin	2000:280	Costa Rica
*Samiriamorpha*	*grace*	Erwin & Geraci	2008:90	Perú
*Tachysbembix*	*sirena*	Erwin	2004:4	Costa Rica
*Tachysbembix*	*wendyporrasae*	Erwin	2004:6	Costa Rica
*Valeriaaschero*	*flora*	Erwin	2004:15	Costa Rica; Panamá
*Valeriaaschero*	*nigrita*	Erwin	2004:16	Costa Rica
*Xenaroswelliana*	*deltaquadrant*	Erwin	2007:564	Brazil
*Xystosomus*	*convexus*	Erwin	1973:29	Brazil
*Xystosomus*	*impressifrons*	Erwin	1973:32	Brazil
*Xystosomus*	*laevimicans*	Erwin	1973:31	Brazil
*Xystosomus*	*laevis*	Erwin	1973:30	Brazil
*Xystosomus*	*niger*	Erwin	1973:32	Brazil
*Xystosomus*	*paralaevis*	Erwin	1973:30	Brazil
*Xystosomus*	*tholus*	Erwin	1973:34	Brazil
*Yasunimorpha*	*piranha*	Erwin & Geraci	2008:88	Ecuador

**Figure 1–12.**
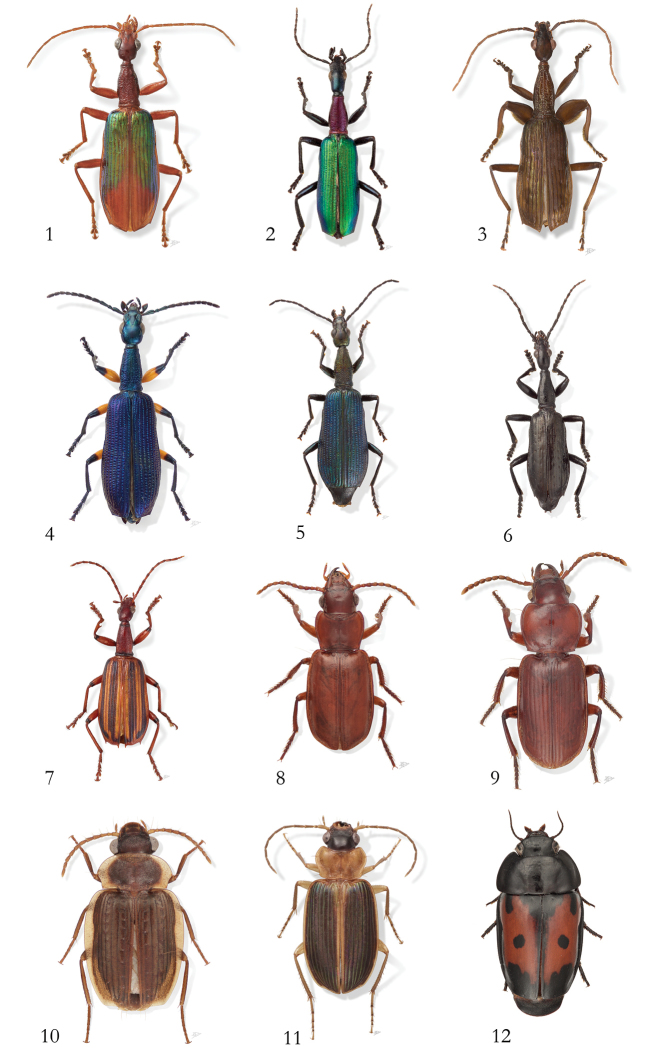
Photos of species described by Terry Erwin. **1** *Agra katewinsletae* Erwin, 2002 **2** *Agra vation* Erwin, 1983 **3** *Agra schwarzeneggeri* Erwin, 2002 **4** *Agra grace* Erwin, 2010 **5** *Agra risseri* Erwin, 2010 **6** *Agra liv* Erwin, 2002 **7** *Agra vate* Erwin, 1986 **8** *Mizotrechus bellorum* Erwin, 2011 **9** *Mizotrechus edithpiafae* Erwin, 2011 **10** *Hybopteroides karolynae* Erwin & Ball, 2012 **11** *Badister amazonus* Erwin & Ball, 2011 **12** *Guyanemorpha spectabilis* Erwin, 2013. Photos: Karolyn Darrow.
